# Diagnostic stewardship – optimization of superficial wound swab cultures can reduce the environmental impact of the microbiology laboratory

**DOI:** 10.1099/acmi.0.000977.v3

**Published:** 2025-09-03

**Authors:** Callum Goolden, Robert J. Shorten

**Affiliations:** 1 UKHSA, Manchester, England, UK; 2 Department of Microbiology, Lancashire Teaching Hospitals NHS Foundation Trust, Lancashire, UK; 3 Queen Mary University of London, London, UK; 4 University of Manchester, Manchester, UK

**Keywords:** diagnostic stewardship, environmental sustainability, laboratory diagnostics, superficial swab, wound swab culture

## Abstract

**Introduction.** Optimization of diagnostic testing is essential for the sustainable delivery of laboratory services. To date, little consideration has been given to the potential benefits of diagnostic stewardship to laboratories looking to reduce their environmental footprint.

**Hypothesis.** Implementing a pre-analytical diagnostic stewardship intervention for the testing of superficial wound swabs would result in a measurable reduction in the environmental footprint of the microbiology laboratory.

**Aim.** To assess the consequential impact of a diagnostic stewardship intervention on test volume, carbon footprint and quantity of non-recyclable plastic waste generated.

**Methodology.** Superficial wound swabs received in the absence of clinical details suggestive of active skin and soft tissue infection were rejected by the laboratory. The carbon footprint of testing was estimated using Publicly Available Specification 2050:2011 methodology in a cradle-to-grave, attributional life-cycle assessment within a defined system boundary. The mass of laboratory plastic waste was calculated through the accurate weighing of associated laboratory consumables.

**Results.** The intervention resulted in a reduction of 35.77 kg CO_2_e and 9.06 kg of unrecyclable plastic waste over an 8-day period without measurable patient harm.

**Conclusion.** This study demonstrates, in relation to specific testing pathways, that the optimization of microbiology laboratory diagnostic testing can result in a reduction in greenhouse gas emissions and non-recyclable plastic waste generation without negatively impacting patient care.

**Impact Statement**

To the best of the authors’ knowledge, this paper represents the first effort to evaluate the environmental impact of a diagnostic stewardship intervention in clinical microbiology. With an ever-increasing pressure being placed on the National Health Service to reduce its environmental impact in line with key commitments, diagnostic laboratories will be forced to adapt their practices and embrace more sustainable service delivery. This paper provides clear evidence of the value of diagnostic stewardship in reducing the environmental impact of laboratory testing and provides suggestions for future implementation methods.

## Data Summary

The UK Government Greenhouse Gas Conversion Factors for Company Reporting (2023 version 1.1) was used in the calculation of greenhouse gas emissions associated with superficial swab laboratory processing (URL: https://view.officeapps.live.com/op/view.aspx?src=https%3A%2F%2Fassets.publishing.service.gov.uk%2Fmedia%2F649c5340bb13dc0012b2e2b6%2Fghg-conversion-factors-2023-condensed-set-update.xlsx&wdOrigin=BROWSELINK).

The authors confirm all supporting data, code and protocols have been provided within the article or through supplementary data files.

## Introduction

Climate change is increasing the frequency and severity of many extreme weather and weather-related events. Increasing average global temperatures are driving shifts in the geographic range of climate-sensitive infectious diseases, affecting food and water security, worsening air quality and damaging socioeconomic systems [1]. Healthcare is a significant contributor to environmental damages. Globally, the carbon footprint of healthcare has been estimated to account for 4.4% of total carbon dioxide equivalent (CO_2_e) emissions [2]. In the UK, the National Health Service (NHS) alone is responsible for 5% of the total national CO_2_e emissions [3]. In October 2020, the NHS became the first health service to commit to reaching carbon ‘Net-Zero’, committing to meet this target for emissions directly under NHS control by 2040 and those for which it can influence by 2045 [4]. Net Zero has since been embedded into legislation via the Health and Care Act 2022.

Microbiology laboratories are highly energy intensive. Facilities utilize specialist diagnostic equipment, incubators, safety cabinets, autoclaves and cold storage. Additionally, heating, ventilation and air conditioning (HVAC) systems employed to sufficiently control humidity and temperature are associated with significant energy consumption [5]. Compounding the aforementioned, microbiology laboratories increasingly operate over extended hours, often offering 24 h service provision, meaning that there is no scope for end-of-day shutdown, powering down of equipment and dimming or turning off lighting. Sample transportation to centralized laboratories also likely generates considerable CO_2_e emissions. Laboratories use large volumes of non-recyclable plastic, and the environmental impact of clinical waste disposal is significant [6,7]. In addition, microbiology waste often requires additional processing pre-disposal. Sterilization of infectious waste by autoclave has been estimated to result in the generation of 338 kg CO_2_e per tonne of waste in addition to the CO_2_e emissions associated with standard disposal via low- or high-temperature incineration or landfill [8].

### Diagnostic stewardship

Clinical diagnostic laboratories in the UK process over 1 billion tests each year, costing in excess of £2.2 billion [9]. NHS laboratories in England processed a total of 30 million samples for microbiological analysis in 2022 alone (NHSE, personal correspondence). It has been shown that the volume of tests performed by diagnostic laboratories in England is increasing by 10% year-on-year [10]. However, it is recognized that not all diagnostic tests will add value to the clinical care and management of patients. In a 2015 meta-analysis, overutilization during initial testing was estimated to occur in 43.9% of cases [11], highlighting clear misuse of diagnostic services worldwide. The ordering of tests in regular intervals or on a routine basis may lead to further unnecessary diagnostic and therapeutic procedures resulting in patient harm by the way of unnecessary stress and potential side effects of subsequent medications or complications from surgical interventions.

Diagnostic stewardship has been suggested as a potential solution to the issue of overutilization or improper use of clinical diagnostic services [12]. The term has been defined as ‘modifying the process of ordering, performing, and reporting diagnostic tests to improve the treatment of infections and other conditions’ [13]. More simply, diagnostic stewardship refers to a process that ensures the right test is performed on the right patient, at the right time to ensure the appropriate action. Diagnostic stewardship interventions are split into pre-analytic, analytic and post-analytic stages. Given that improving test utilization can lead to a reduction in testing volume, diagnostic stewardship may have a key role to play in helping laboratories reduce their environmental footprint.

### Diagnostic stewardship for superficial swab cultures

Preliminary scoping data showed that 14,204 wound cultures were processed by Lancashire Teaching Hospitals NHS Foundation Trust Microbiology Department in 2022 alone. Superficial swabs were found to be the second most frequently processed sample, second only to urine, in the bacteriology section.

Anecdotally, it is felt that many wounds and chronic ulcers are swabbed in the absence of clinical signs of active infection. The interpretation of any growth seen from superficial sites is difficult due to colonization of the skin surface by organisms that can be both considered as normal microbiota, but also as primary pathogens should there be a compromise in the healthy skin barrier. This overutilization of laboratory services generates significant volumes of work, unnecessary CO_2_e emissions, large volumes of unrecyclable plastic waste and the potential for inappropriate antibiotic prescribing based on laboratory findings.

## Methods

### Objective

We sought to evaluate the environmental impact of implementing a pre-analytical diagnostic stewardship intervention for superficial swab cultures. Primary outcomes included the consequential impact on total test volume, the associated carbon footprint of laboratory analyses and the quantity of unrecyclable plastic waste generated.

### Study setting

The study was conducted within the diagnostic microbiology laboratory of Lancashire Teaching Hospitals NHS Foundation Trust (LTH), a large secondary care centre in the North West of England. The laboratory serves two acute general hospitals, with ~920 inpatient beds cumulatively, and a network of primary care centres serving a population of ~1.5 million. The bacteriology laboratory performed 287,335 analyses in 2022, including 149,058 swabs, of which 14,204 were from superficial wounds.

### Study design

We performed a pre-post-comparative study, with an initial 8-day pre-intervention period to obtain baseline data followed by an 8-day intervention period in which all superficial wound swabs received by the microbiology laboratory meeting pre-defined inclusion criteria were assessed. The intervention was applied during routine working hours (08:30–17:00, Monday–Friday). Samples processed out of hours or over the weekend were not included.

#### Diagnostic stewardship intervention

The intervention was implemented within the pre-analytical portion of the local diagnostic pathway for superficial wound swab microbiological investigations.

Requests for culture of superficial wound swabs were screened at receipt. Pre-defined exclusion criteria (Table 1) were applied, and excluded specimens were processed as standard. The quality of the clinical details accompanying the remaining specimens was then assessed.

**Table 1.** Intervention period exclusion criteria. Current antimicrobial therapy was elucidated by accessing the electronic prescription charts, primary care record and written information accompanying the request

Exclusion criteria
Resistant organism screening – includes methicillin-resistant *Staphylococcus aureus*, vancomycin-resistant enterococci and carbapenemase-producing organisms.
Deep tissue samples
Swabs from patients with burns
Swabs from patients <16 years old
Request specifically made by an infection specialist.
Samples from patients prescribed antimicrobial therapy for skin and/or soft tissue infection as part of the current care episode, e.g. listed as an indication for treatment within ±24 h of sample collection.

Requests with clinical details suggestive of skin and/or soft tissue infection (Table 2) were processed as routine. Low-quality samples with clinical details deemed not suggestive of active infection were rejected. A second opinion from a member of the clinical microbiology team was sought in the presence of any uncertainty.

**Table 2.** Appropriate clinical features justifying the collection of superficial wound swabs. The presence of one or more of these clinical details prompted routine processing of the sample

Clinical details criteria: ‘suggestive of active skin and/or soft tissue infection’.
Localized redness (erythema)
Localized pain
Localized heat
Localized oedema (swelling)
Cellulitis
Abscess/pus
Discoloured, offensive and purulent discharge
Malodour
Delayed healing (or failure to heal) compared to normal rate for site and condition
Discolouration of wound bed
Friable granulation that bleeds easily/over granulation
Pocketing/bridging at the base of the wound
Wound breakdown/enlargement

For rejected specimens, an immediate electronic result was issued stating: ‘Indication for swab unclear due to lack of or inadequate clinical details. Sample not processed. If you feel that this sample should be processed, please email xxxxxxxxxxxx@lthtr.nhs.uk within 72 h of specimen collection’. Inclusion of this report comment enabled the requesting clinician to contact the laboratory for urgent processing if required to inform management decisions. Swabs were stored for a period of 7 days at 2–8 °C to enable subsequent processing as required.

#### Carbon footprint calculations

**Fig. 1.**
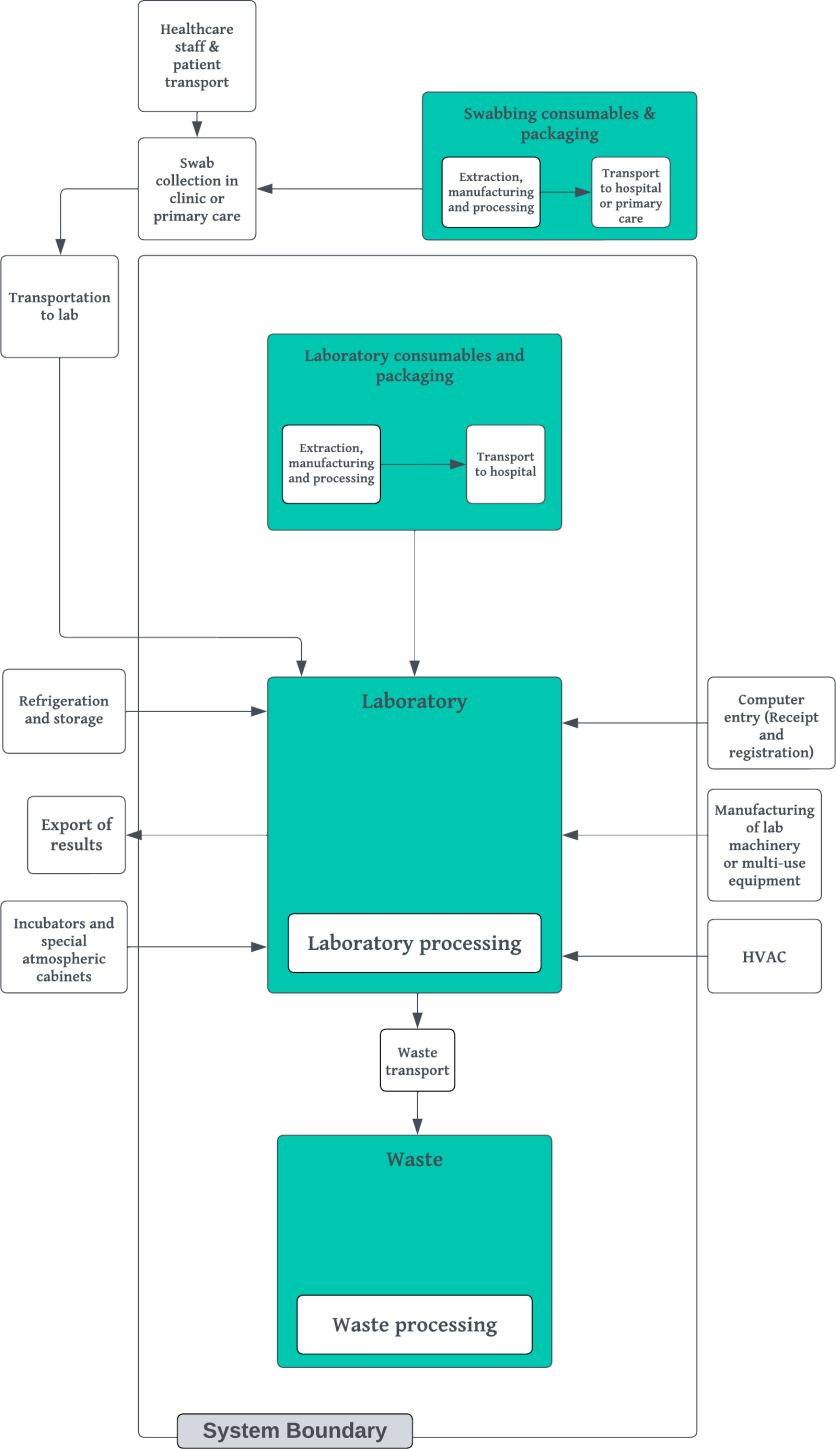
LCA system boundary. Elements and processes within the boundary were accounted for, and the attributable carbon dioxide equivalent was calculated as part of the analysis.

The carbon footprint of the laboratory testing of superficial wound swabs was estimated using PAS 2050:2011 methodology [14]. PAS 2050:2011 addresses the single impact category of global warming. It does not account for other environmental impacts such as acidification, eutrophication, toxicity and biodiversity loss. The analysis is a cradle-to-grave, process-driven, attributional Life Cycle Assessment (LCA) within a strictly defined system boundary (Fig. 1). Analysis includes emissions relating to the use of laboratory consumables and subsequent disposal of laboratory waste in the processing of superficial swabs.

Processes upstream of the intervention, including the manufacture and use of swabbing consumables, and travel emissions from patients, staff and sample transportation to the laboratory, were deemed out of scope. Emissions associated with the operation of premises, including HVAC, refrigeration and storage and sample incubators, were also excluded, as these systems or appliances operate continuously irrespective of the number of samples processed. As per PAS 2050:2011 methodology, emissions associated with the manufacture of capital goods were excluded. Additional inputs were excluded from the analysis due to difficulties in calculation and/or published results showing their negligible impact on carbon calculations. Namely, these included emissions associated with energy use from barcode printing and the distribution of culture media by the automated plate organization system.

For laboratory consumables and packaging, calculations cover the extraction, primary processing and transportation of materials to the point of use. The use of virgin as opposed to recycled plastic items was assumed throughout. Emissions were apportioned by the weight of each material contained within a given consumable item. For packaging, the proportion per used item was calculated to encompass the total weight in subsequent calculations. When calculating emissions relating to end waste disposal, initial sterilization of infectious waste by autoclave prior to disposal by combustion was factored in. For recycling and combustion (with energy from waste), the emission factors used consider transport to an energy recovery or materials reclamation facility only, as per the GHG Protocol guidelines. Total CO_2_e values for the intervention period accounted for variation in the number of culture media used by the laboratory depending on the site of the wound. The approximate annual CO_2_e was estimated using laboratory testing figures from 2022.

#### Data sources

Emission factors were obtained from the UK GHG Conversion Factors for Company Reporting database [15]. Where factors were unavailable, emission factors were obtained from other published sources [8].

Primary activity data were obtained for laboratory consumable items and associated packaging. Small plastic items, e.g. Petri dishes (empty) and transport bags weighing less than 200 g, were measured using a Kern PFB200-3 analytical balance (±0.001 g) (Sigma-Aldrich, St. Louis, USA). Heavier shipping packaging (<3 kg) was measured using Salter ARC Digital Kitchen Scales (Salter, Manchester, UK). The material composition of consumable products was obtained via publicly available data or via direct email correspondence with suppliers.

#### Plastic waste

All plastic items used in the laboratory processing of superficial wound swabs were identified from the process map (Data S1, available in the online Supplementary Material). For the intervention period, the total number of each item was calculated. For most, this equated to one per test, but others varied; for example, the number of plates used was dependent on the laboratory coding, where a greater number of different culture media were inoculated for the isolation of a broader range of pathogenic organisms.

The total mass was a sum of items multiplied by their respective individual mass. Approximate yearly plastic savings were estimated by applying the percentage rejection rate from the 8-day intervention to 2022 wound swab testing figures at Lancashire Teaching Hospitals. The percentage positivity rate, defined as organisms with reported antimicrobial susceptibility results, was used to estimate the additional weight of plastic associated with susceptibility testing of significant isolates.

#### Patient impacts

For clinical specimens not processed during the intervention period, and for those rejected as per protocol but subsequently tested upon clinician request, antibiotic prescriptions were reviewed retrospectively to determine if the patient was actively managed for a skin and soft tissue infection (SSTI). For specimens excluded due to the presence of an active antibiotic prescription for an SSTI, the culture positivity rate and the change in antimicrobial management were evaluated.

## Results

### Diagnostic stewardship intervention

#### Intervention period

The total number of swabs collected, mean samples per day, appropriateness of clinical details, percentage of swabs excluded and percentage of swabs rejected as per protocol were largely comparable between the pre-intervention (baseline) group and the study intervention group (Tables3  4).

**Table 3.** Total samples (n) received in the pre-intervention period, grading of clinical detail appropriateness and breakdown of specimens meeting pre-determined exclusion criteria. The table does not illustrate the number of samples rejected in the intervention arm (Table 2)

Pre-intervention period:	
**Superficial wound swabs received**	392
Daily mean/sd	49/9.29
Clinical details suggestive of active SSTI	62 (16%)
Clinical details provide some clinically useful information but provide no clear indication of active SSTI	156 (40%)
Clinical details absent or provide little to zero clinically useful information	174 (44%)
**Wound swabs meeting exclusion criteria**	182 (46%)
*Resistance screen*	1 (<1%)
*Deep tissue*	2 (1%)
*Patients with burns*	17 (9%)
*<16* years	16 (9%)
*Infection specialist request*	0 (0%)
*On antibiotics for SSTI indication at time of receipt*	146 (80%)

**Table 4.** Total samples (n) received in the intervention period, grading of clinical detail appropriateness and breakdown of excluded specimens meeting pre-determined criteria. The table does not illustrate the number of samples rejected in the intervention arm (Table 2)

Intervention period:	
**Superficial wound swabs received**	407
Daily mean/sd	51/15.61
Clinical details suggestive of active SSTI (n)	74 (18%)
Clinical details provide some clinically useful information but provide no clear indication of active SSTI (n)	141 (35%)
Clinical details absent or provide little to zero clinically useful information (n)	192 (47%)
**Wounds excluded from study**	210 (52%)
*Resistance screen*	3 (<1%)
*Deep tissue*	3 (1%)
*Patients with burns*	19 (9%)
*<16* years	18 (9%)
*Infection specialist request*	2 (<1%)
*On antibiotics for SSTI indication at time of receipt*	165 (79%)

**Fig. 2.**
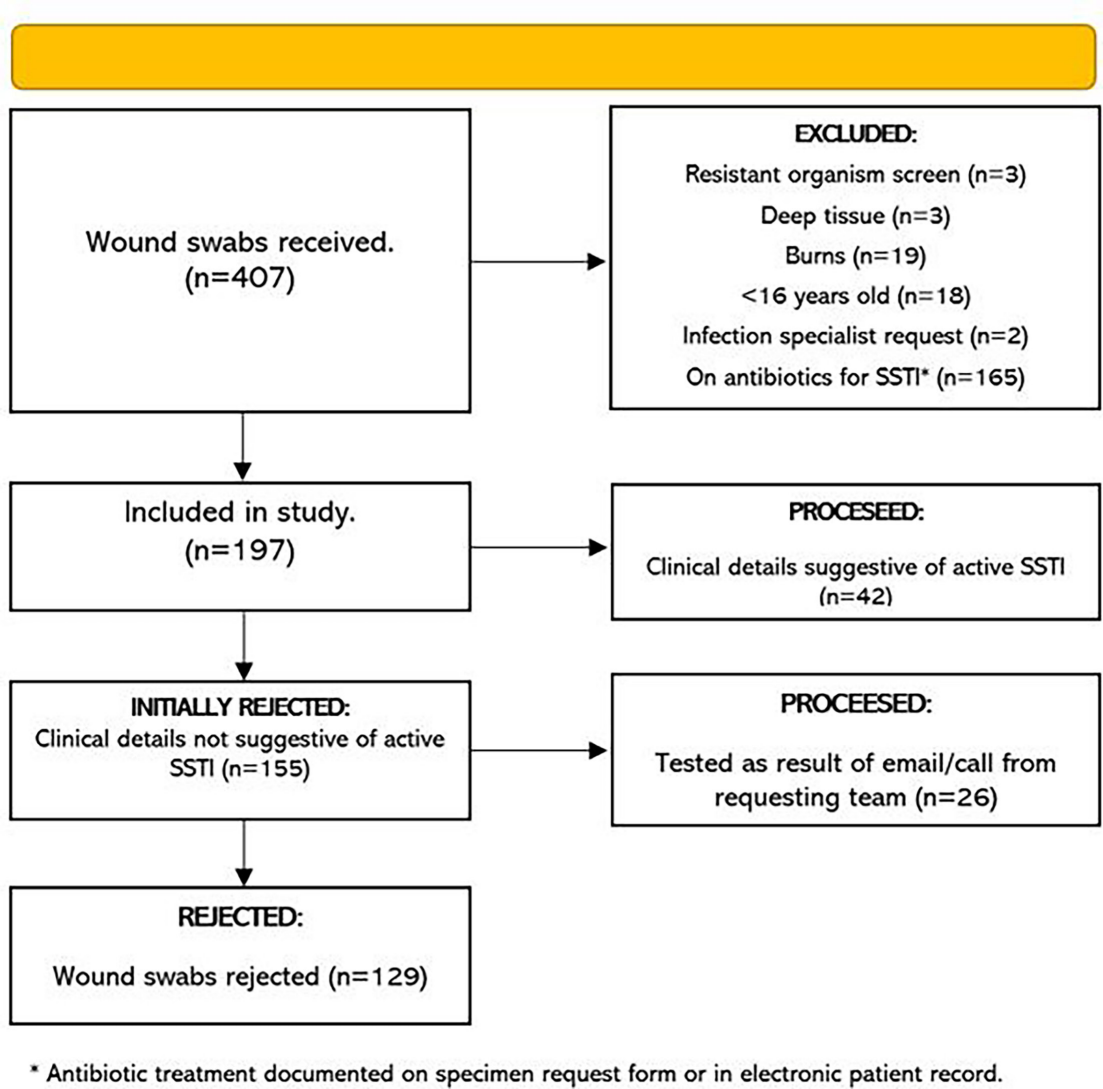
Intervention period flow diagram.

Of a total of 407 superficial wound swabs received during the intervention period, 197 (48.4%) met the inclusion criteria. Of this sample, 42 specimens (27.10%) had clinical details suggestive of active SSTI and were thus processed as per laboratory standard operating procedures. The remaining 155 swabs (72.9%) were initially rejected as per protocol (Fig. 2).

The laboratory received a total of 29 calls or emails from requesting teams providing additional justification for testing which led to the subsequent processing of 26 of the previously rejected swabs. At the end of the intervention period, a total of 129 samples had been rejected and were not processed by the laboratory. This represents 31.7% of the total swabs received during this period and 65.8% of those included in the investigation. Only 16.8% of swabs initially rejected at screening were processed as a result of clinician follow-up with the laboratory, meaning that 83.2% of rejected swabs did not prompt any clinician follow-up.

### Carbon footprint analysis

**Fig. 3.**
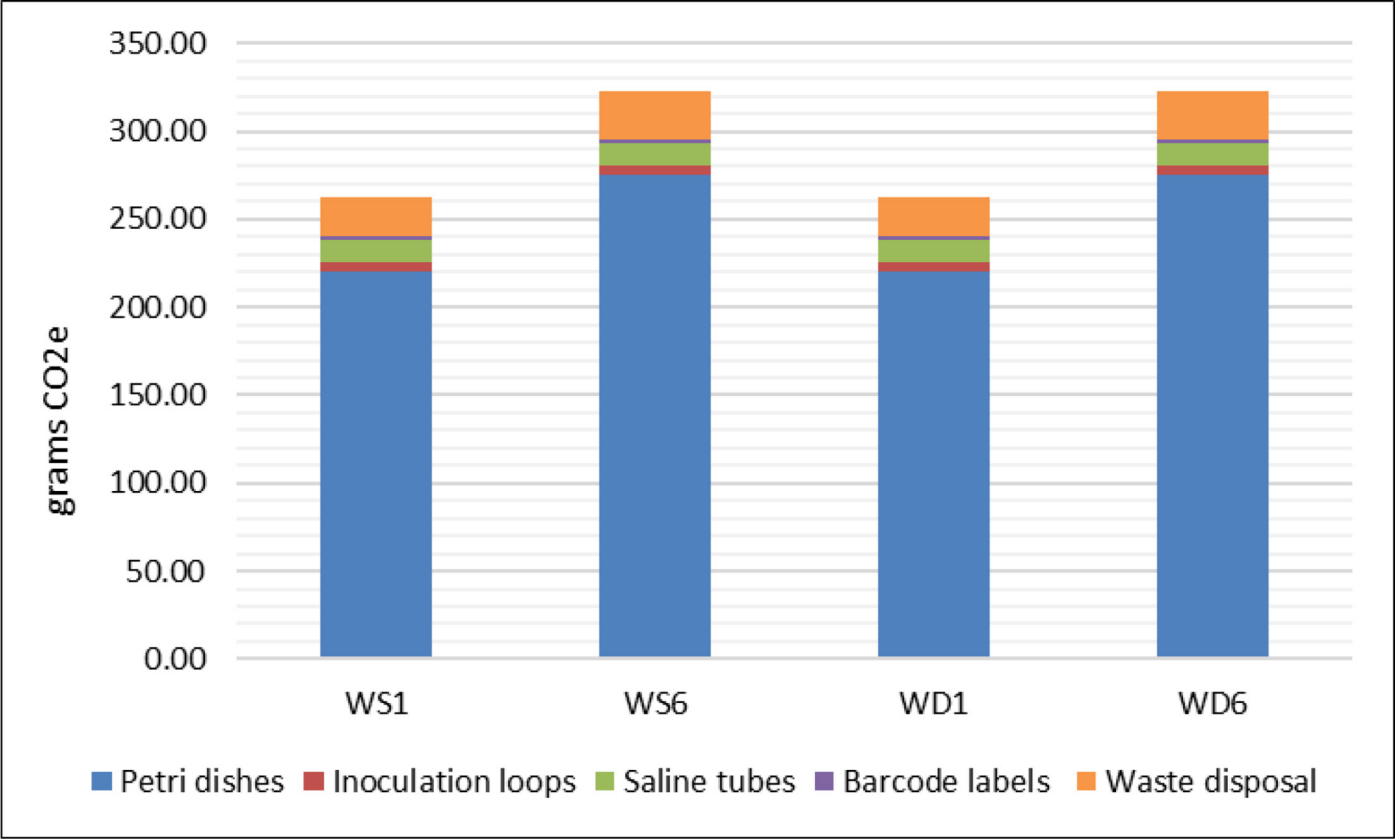
Sources of GHG emissions (gCO_2_e) associated with the laboratory processing of superficial wound swabs. Laboratory codes refer to the choice of growth media inoculated. WS1=‘dirty sites’, e.g. below the waist – blood agar (O_2_), CLED, CAP and NEO (+Met). WS6=‘dirty sites’ with indication for fungal investigation (WS1+additional SAB plate). WD1: swab from ‘clean site’, e.g. above waist – blood agar (O_2_), blood agar (ANO_2_), CLED and NEO (+met). WD6=‘clean sites’ including indication for fungal investigation (WD1+additional SAB plate).

Opting to process the 129 superficial wound swabs rejected as part of the intervention arm would have resulted in the generation of 35.77 kg CO_2_e. Applying UK Government GHG Conversion Factors [15], this is equivalent to driving a small petrol car for 157.84 miles. For individual test requests, depending on laboratory coding, this works out as between 262.79 and 323.01 gCO_2_e per test performed (Fig. 3), with the majority of the carbon footprint of superficial wound swab processing attributable to the production of plastic Petri dishes.

Applying this to 2022 testing figures, the implementation of the intervention over a 12-month period would result in an estimated annual 3,952.89 kg CO_2_e reduction. This is equivalent to driving a small petrol engine car ~17,444 miles [15].

### Plastic waste

The intervention resulted in an estimated reduction in plastic waste generation of 9.06 kg over the 8-day intervention. Extrapolated across a 12-month period, using 2022 testing figures as a guide, this would equate to 311 kg of unrecyclable plastic waste prevented as a result of a simple pre-analytical measure.

### Patient impact

Of the 26 requests initially rejected in the intervention arm, but subsequently tested as a result of follow-up communication, 22 out of 26 resulted in no documented change in clinical management. For the remaining 4 out of 26, the electronic patient notes were not accessible. Three out of twenty-six did not have documented antibiotic prescriptions at the time of screening but were subsequently found to be on antibiotics for SSTI and thus would have been included as per the exclusion criteria.

Of the 165 swabs excluded from the study due to documented antibiotic treatment for SSTI, 78 grew clinically significant organisms. Of these, 61 resulted in no change in antimicrobial management within 72 h of report generation. Only 17 (21.79%) results led to a change in antimicrobial management within this timeframe.

## Discussion

Through the implementation of a pre-analytical diagnostic stewardship intervention for superficial swab culture and sensitivity testing, we were able to demonstrate that the optimization of microbiology laboratory diagnostic testing can result in a reduction in GHG emissions and unrecyclable plastic waste generation. The study supplements previous work assessing the carbon footprint of clinical laboratory analyses [6,7, 16,19].

Over an 8-day intervention, a total of 129 superficial swabs were rejected and not processed. This represented 31.7% of the total superficial wound swabs received during this period, and 65.8% of swabs included in the study. Anecdotally, it is widely accepted that a large proportion of superficial wound swabs are collected unnecessarily, and the lack of follow-up requests to process rejected samples provides evidence to support this claim. In the absence of clinical signs of infection or epidemiological reasons, superficial wounds should not be cultured if there is no indication for antimicrobial therapy. The processing of swabs from infected wounds aids only to rationalize or discontinue empirical antibiotic treatment of clinically infected wounds [20]. Previous work by a Canadian group using the Q-score quality metric for grading superficial swabs demonstrated that 58% of swabs were of low quality [21], although not directly comparable, the proportion of swabs regarded as low value and rejected as part of our intervention was not dissimilar in magnitude.

The study clearly demonstrates that implementing diagnostic stewardship initiatives has the potential to significantly impact the environmental impact of the microbiology laboratory. The 8-day intervention resulted in the prevention of 35.77 kg CO_2_e generation and the production of 9.06 kg of unrecyclable plastic waste, both directly attributable to laboratory operations. The approximated annual savings of 3,952.89 kg CO_2_e and 311.08 kg plastic, respectively, are not insignificant for an intervention primarily designed to improve the quality of laboratory analysis and subsequent patient management. This study represents the first attempt to quantify the carbon footprint of superficial wound swab culture and sensitivity analysis. The GHG emission figures calculated supplement earlier, more granular LCAs of other laboratory diagnostic tests such as full blood counts, urea and electrolytes, arterial blood gases, C-reactive protein and urinalysis [6,18]. As seen with urinalysis, our findings demonstrate that the majority of the GHG emissions associated with sample processing in the microbiology laboratory are associated with the initial extraction, manufacturing and transportation of laboratory consumables, namely, the polystyrene Petri dishes used to house agarose growth media for bacterial culture.

Despite recording reductions in total test numbers, GHG emissions, waste generation and costs, no potential patient harm was identified. The laboratory was contacted (phone or email) on 29 separate occasions regarding a total of 43 clinical specimens. Some of these calls facilitated a degree of education on the correct utilization of superficial swabs in the management of patients with suspected SSTI. One clinical team called to enquire as to why the laboratory had rejected 13 wound swabs taken from the pin sites from a patient with an external fixation device. When queried on the nature of the site, it was elucidated that only a single pin site was ‘possibly infected’; however, which pin, and more importantly which swab this mapped to, was unknown. Importantly, 83.2% of rejected samples resulted in no follow-up from clinical teams. For these samples, it may be that the clinician agreed with the decision to not process or that workload burden resulted in a limited time to fully review less critical results. Of the 26 specimens initially rejected and subsequently re-tested following clinician follow-up, none resulted in a documented change to clinical management with 7 days of release of the rejection comment. This may suggest a lack of understanding of the clinical utility of superficial swab culture results amongst clinicians locally but could also be due to patient-led decisions regarding their own care whereby they choose not to re-present for follow-up. Only 78 of the 165 samples from patients on active treatment grew significant isolates, with only 17 findings resulting in a change in antibiotic or clinical management indicating either a lack of result review or a limited number of occasions where rationalization or escalation of antibiotics is required.

Although our intervention focussed on laboratory processes following the receipt of samples, it is clear that the greatest opportunity to reduce the environmental impact of testing is by preventing unnecessary requests at the point of sampling. The collection of wound swabs in the hospital or the community requires the use of additional consumable items, including but not limited to the swab itself, paper request forms and transport bag(s). In addition, during sampling, healthcare staff may wear personal protective equipment, such as nitrile gloves, and use bottled saline to irrigate and disposable sterile wipes to clean the wound surface. The additional contribution of these steps, including the transportation of samples from the point of collection to the laboratory, is likely to contribute additional GHG emissions, as well as additional unrecyclable waste material on top of the items used during laboratory investigations. Ultimately, the presence or absence of potentially pathogenic organisms from such samples cannot categorically rule in or rule out infection. The diagnosis of SSTI is one made largely on clinical grounds, and the sampling of non-infected wounds should be discouraged.

Despite successful application during this trial, the intervention of rejecting unsuitable samples on the basis of inappropriate clinical details is not particularly suitable or translatable to routine practice. Screening of clinical details and antibiotic prescriptions at receipt is time-consuming (1–2 min per sample) and would require the availability of a suitably trained clinical or laboratory professional at all times. Whilst technically feasible, this is not an effective use of resources and would potentially negate any potential financial savings. A more viable alternative would be to utilize a bespoke computerized clinical decision support (CCDS) tool embedded within the hospital and primary care test requesting systems which would only permit ordering of tests when responses to questions satisfy the algorithm that the patient has a genuine need for sampling. CCDS tools have already been successfully used in the diagnostic stewardship of *Clostridioides difficile* infections [22,23], but appropriateness and effectiveness of such a tool for superficial wound swabs have yet to be assessed.

### Limitations and caveats

The study data come with several significant limitations and caveats. Firstly, the intervention only covered a short window (8 days). Swabs received and processed over weekends and bank holidays were not evaluated, making extrapolation of results to produce annual estimates for total GHG emissions and plastic waste prone to inaccuracy. The intervention period also coincided with a period of industrial action by junior doctors, which may have influenced the frequency of wound swab collection. A significant caveat is that clinical leads and general practitioners were notified of the study in advance. In an attempt to ameliorate any potential bias, a significant buffer period was allowed, and observationally, this does not appear to have impacted on the quality of the clinical details provided alongside requests. For the cohort of patients on antibiotics, it was often a clinical judgement call for community-based patients on whether the antibiotics listed in their record were prescribed for an active SSTI or for an alternative reason due to the lack of indication listing in the GP record. Conversely, at screening, the investigation was still reliant on the provision of appropriate clinical details where testing was appropriate, which may have unduly led to the rejection of appropriately collected samples.

For the calculation of the GHG emissions and plastic waste associated with the intervention, calculations assumed the use of a single saline suspension tube and agar plate per clinically significant isolate grown. This is unlikely to have been the case uniformly, as many samples will be polymicrobial and isolate more than a single organism of interest. Similarly, the calculation of the contribution of antimicrobial susceptibility testing of significant isolates utilized the percentage of samples from which reportable bacteria were grown. Some specimens may have grown more than a single isolate, thus resulting in an overestimation of percentage positivity. A further methodological limitation is that the impacts of patient travel and sample transportation to the laboratory were not accounted for.

The assessment of patient potential harm was heavily focussed on changes in antibiotic treatment as a proxy for unsuccessfully treated genuine infections. The methodology does not allow for additional potential harms such as the psychological effects of not receiving a result. It is also worth highlighting that the findings relating to patient harm are not generalizable due to the short study duration and small sample size evaluated.

## Conclusions

Optimization of microbiological testing and restriction of low-value requests can reduce the environmental impacts of laboratory analysis, whilst simultaneously increasing the quality and utility of results and reducing costs. However, despite this study demonstrating that the restriction of testing based on clinical details to be promising, the feasibility of having a dedicated laboratory scientist screening all incoming swabs is unlikely to be a viable option for most laboratory services. However, the large proportion of swabs that were rejected and therefore not processed during this study demonstrates that many specimens are not adding clinical value to patient care. This should act as a clarion call to laboratory specialists to review sampling processes and to engage with our service users to ensure that we maximize the quality of such sampling. Since most CO_2_e has already been expended by the time a clinical sample reaches the laboratory, the prevention of inappropriate sampling should be focussed upstream, with clinical users of pathology services, such as with the use of electronic pre-analytic interventions such as CCDS tools, and targeted clinical education may be more impactful.

In terms of future directions for further work, a more granular assessment of the carbon footprint of wound swab investigations is still required. A full LCA of the process, including the contribution of laboratory estate, will provide a more comprehensive assessment of how much GHG emissions are attributable to the process and will provide greater guidance on how we can optimize and reduce the carbon footprint of testing. Following on from this study, a trial looking at the implementation of a CCDS for superficial swab cultures, assessing the impact on test numbers, appropriateness and accompanying environmental impacts, is the logical next step.

## Supplementary material

10.1099/acmi.0.000977.v3 Uncited Supplementary Material 1.

10.1099/acmi.0.000977.v3 Uncited Supplementary Material 2.

## Abbreviations

CCDS: computerized clinical decision support
GHG: Government Greenhouse Gas
HVAC: heating, ventilation and air conditioning
LCA: Life Cycle Assessment
NHS: National Health Service
SSTI: skin and soft tissue infection
