# Supplementary material for: Diagnostic stewardship – optimization of superficial wound swab cultures can reduce the environmental impact of the microbiology laboratory
Source: Access Microbiol. 2025 Sep 3;7(9):000977.v3. doi: 10.1099/acmi.0.000977.v3 (PMC12408188; doi:10.1099/acmi.0.000977.v3)
Supplement: Uncited Supplementary Material 1. [file acmi-7-00977-s001.pdf]

Process Flow:

The wound site is first gently cleansed with sterile water or saline to remove sloughy material. Rayon bud swabs are used to sample the affected area. If the wound is dry, then the end of the swab is moistened in sterile saline prior to swabbing. Under aseptic conditions, samples are collected from the deepest part of the wound, trying to avoid the superficial microflora. The swab is rotated gently across a representative area of the wound, placed into the charcoal (Amies) transport medium containing tube and the lid securely fastened. Sample labels are printed and attached to both sample and paper request form. The specimen is then transported to the laboratory by portering staff orinter-site transport. .

Specimens are sorted, removed from their plastic transport bag, and electronically ordered or receipted by laboratory staff. An adhesive label with a unique laboratory number is affixed to the swab. Requests are coded by medical laboratory assistants in accordance with the site of the wound and any clinical details provided to ensure the appropriate solid growth media is inoculated to aid isolation of clinically significant target organisms. Culture media is labelled and distributed by the Plate Organisation System (POS) and inoculated by hand. The initial inoculum is spread using plastic inoculation loops to facilitate the isolation of discrete bacterial colonies for further work. Inoculated plates are placed in sample incubators for 16-24 hours to stimulate bacterial growth. Biomedical Scientists assess media for growth of significant organisms and perform identification tests and antimicrobial susceptibility testing by disc diffusion or MIC gradient strip testing as appropriate. Results are manually input into the Laboratory Information Management System (LIMS) and are reviewed and validated by a member of the clinical microbiology team before being electronically exported to the service user in the form of a final report. Used plates and inoculation loops are sterilised by autoclave and disposed of by an external contractor.
